# The ghrelin system follows a precise post-natal development in mini-pigs that is not impacted by dietary medium chain fatty-acids

**DOI:** 10.3389/fphys.2022.1010586

**Published:** 2022-09-26

**Authors:** Gaëlle Boudry, Armelle Cahu, Véronique Romé, Régis Janvier, Margaux Louvois, Daniel Catheline, Vincent Rioux, Isabelle Le Huërou-Luron, Sophie Blat

**Affiliations:** ^1^ Institut Numecan, INRAE, INSERM, Univ Rennes, Saint-Gilles-Rennes, France; ^2^ Institut Agro, Rennes, France

**Keywords:** ghrelin, ghrelin-ghrelin O-acyltransferase system, convertase 3, caprylic (C8:0) and capric acid (C10:0), neonate

## Abstract

The ghrelin-ghrelin receptor (GHSR1) system is one of the most important mechanisms regulating food intake and energy balance. To be fully active, ghrelin is acylated with medium-chain fatty acids (MCFA) through the ghrelin-O-acetyl transferase (GOAT). Several studies reported an impact of dietary MCFA on ghrelin acylation in adults. Our study aimed at describing early post-natal development of the ghrelin system in mini-pigs as a model of human neonates and evaluating the impact of dietary MCFA. Suckled mini-pigs were sacrificed at post-natal day (PND) 0, 2, 5, and 10 or at adult stage. In parallel, other mini-pigs were fed from birth to PND10 a standard or a dairy lipid-enriched formula with increased MCFA concentration (DL-IF). Plasma ghrelin transiently peaked at PND2, with no variation of the acylated fraction except in adults where it was greater than during the neonatal period. Levels of mRNA coding pre-proghrelin (GHRL) and GOAT in the antrum did not vary during the post-natal period but dropped in adults. Levels of antral *pcsk1/3* (cleaving GHRL into ghrelin) mRNA decreased significantly with age and was negatively correlated with plasma acylated, but not total, ghrelin. Hypothalamic *ghsr1* mRNA did not vary in neonates but increased in adults. The DL-IF formula enriched antral tissue with MCFA but did not impact the ghrelin system. In conclusion, the ghrelin maturation enzyme PCSK1/3 gene expression exhibited post-natal modifications parallel to transient variations in circulating plasma ghrelin level in suckling piglets but dietary MCFA did not impact this post-natal development.

## Introduction

Ghrelin is a 28 amino acid peptide with pleiotropic effects. This hormone displays potent orexigenic and adipogenic activities (Tschöp et al., 2000), anti-apoptotic effects, especially in the pancreas (Wang et al., 2010), anti-depressant effects in adults (Lutter et al., 2008) and promotes sleep (Steiger et al., 2011). Ghrelin is mainly produced in the stomach and results from preproghrelin maturation within gastric enteroendocrine cells. The cleavage from the precursor form to ghrelin is catalyzed by the convertase 3 (PCSK1/3). Ghrelin can then be acylated by medium chain fatty acids (MCFA), mainly octanoic acid (caprylic acid, C8:0) but also hexanoic acid (caproic acid, C6:0) and decanoic acid (capric acid, C10:0) as revealed by dietary supplementation experiments in mice (Nishi et al., 2005a) and structure-activity analysis (Darling et al., 2015). This acylation reaction is mediated by the ghrelin-O-acyltransferase (GOAT) (Gutierrez et al., 2008; Yang et al., 2008). In the plasma, both acylated and non-acylated ghrelin circulate but only the acylated form is recognized in the central nervous system, where it activates the growth hormone secretagogue receptor (GHSR1a) to exert its biological effects (Kojima et al., 1999). Ghrelin action upon food intake and energy metabolism regulation can also be mediated by the vagus nerve, as recently reviewed by Perello et al. (2022)

The post-natal development of the ghrelin system in humans is not fully described yet. Ghrelin is present at birth since immuno-reactive (IR) non acylated–ghrelin cells have been observed in the fetal stomach, duodenum, pancreas, and lung from the 10th week of gestation in humans (Volante et al., 2002). Moreover, ghrelin has been detected in the cord blood at birth (Bellone et al., 2004). Several clinical studies indicate that ghrelin plasma level increases after birth, peaking during the first 2 years of life (Soriano-Guillén et al., 2004; Savino et al., 2005, de Fluiter et al., 2021), then decreasing until adulthood (Whatmore et al., 2003; Wilasco et al., 2012). Interestingly, the decrease in plasma ghrelin after 2 years of age was mainly attributed to the non-acylated form but not the acylated one (Wilasco et al., 2012). A post-natal increase in plasma and stomach ghrelin levels (both total and acylated forms) during neonatal life was also observed in mice (Nishi et al., 2005b; Steculorum et al., 2015). This post-natal increase in ghrelin plasma paralleled the increase in preproghrelin gene (*ghrl*) expression in the stomach until weaning at post-natal day (PND) 21. Yet, *ghrl* mRNA levels decreased afterwards in adult mice while ghrelin plasma levels remained stable (Liu et al., 2002). In rats, the density of ghrelin–IR and *ghrl* mRNA containing cells increases with post-natal age, even after weaning and up to 8 weeks of age (Sakata et al., 2002).

Whether plasma ghrelin variation during the neonatal period is important for the infant development is not fully understood. In preterm infants, one study reported that ghrelin secretion was not different before and after a meal in 4d-old neonates (Bellone et al., 2004). Likewise, cord blood ghrelin levels did not correlate with total milk intake during the first 7 days of life in bottle-fed neonates (James et al., 2007). Thus, the classical “hunger hormone” role of ghrelin might not be fully functional during the first days of post-natal life (Savino et al., 2012). Interestingly, Steculorum et al. (2015) established in mice that ghrelin signaling exerts an organizational effect on neural projections from the hypothalamus during early post-natal development, thus not only playing a role as central nervous system signal but also in neural development

Considering the role of the establishment of eating behavior regulatory pathways in infancy on later risk of obesity and associated disease development (Carnell and Wardle, 2008), there is a need for a better understanding of the mechanisms and factors influencing the post-natal development of the ghrelin system. Among influencing factors, perinatal diet may be at play. Indeed, several studies reported lower levels of plasma ghrelin in breast-fed compared to bottle-fed infants (Savino et al., 2005; de Fluiter et al., 2021), although discrepancies exist (Savino et al., 2006). Animal models also indicate that perinatal diet during gestation and/or lactation can modify the ghrelin system during the neonatal period or later in life, either at the plasma (Desai et al., 2007; Szeto et al., 2008; Jahan-mihan et al., 2011; Fuente-Martín et al., 2012; Collden et al., 2015; Słupecka et al., 2016; Sideratou et al., 2018; Jahan-mihan, 2019; Kisioglu and Nergiz-Unal, 2020; Sun et al., 2020) or the central level (Yousheng Jia et al., 2008; Prior et al., 2014; Collden et al., 2015; Maldonado-Ruiz et al., 2019; Rossetti et al., 2020; Sun et al., 2020). Yet, the impact of perinatal diet at the stomach level has been less described (Collden et al., 2015; Du et al., 2015; Sun et al., 2020). Several data also suggest that dietary C8:0 and GOAT activity regulate acylated ghrelin production, circulating concentration and functions in rats (Lemarié et al., 2018), although this has been recently challenged (Kaur et al., 2020). Increased circulating acylated ghrelin after orally ingested MCFA has also been observed in other species like chicken (Yamato et al., 2005) and fishes (Kaiya, 2013), suggesting a conserved mechanism. Dietary MCFA can be absorbed in the stomach (Perret, 1980; Lemarié et al., 2015, 2016) and could participate in ghrelin acylation (Nishi et al., 2005a), although endogenous MCFA production through β-oxidation of long chain fatty acids also contribute to ghrelin acylation Milk is the unique source of nutrients for the neonate. Interestingly, breast-milk is rich in MCFA (Ahmed et al., 2021), implying that neonates receive a fair amount of MCFA during the neonatal period compared to other periods of life. We hypothesized that dietary MCFA during the neonatal period play a role in the development of the ghrelin system. Our objective was therefore to first thoroughly study the post-natal development of the different components of the ghrelin system in suckling animals and second, to evaluate the effect of dietary MCFA on the ghrelin system using infant formulas with low and high levels of MCFA. We chose to use piglets, an animal model close to humans in terms of neonatal development and gastro-intestinal physiology (Roura et al., 2016) to conduct this study. Although ghrelin acylation by dietary MCFA has not been formally demonstrated in pig so far, the conservation of the ghrelin structure, and especially that of the acyl-modification regions, among vertebrate species (Kojima et al., 2007) was a strong argument to use this model.

## Materials and methods

### Animal experiment

Animal procedures were performed in accordance with French law and approved by the Comité Rennais d’Ethique en Expérimentation Animale and by the Ministère de l’Enseignement Supérieur et de la Recherche (APAFiS#11014-2017080512029744 v2). Two animal experiments were conducted separately: the first one investigated the evolution of ghrelin-related parameters in suckled mini-pigs and adult ones at different ages while the second one investigated the impact of dietary MCFA on ghrelin-related parameters at one post-natal age (PND 10). In the first experiment, Yucatan suckling mini-pigs from the Unité Experimentale Physiologie et Phénotypage Porcins (UE3P INRAE, Saint-Gilles) were sacrificed at PND 0, 2, 5, and 10. Thirty-five piglets (18 males and 17 females) were selected at birth based on their birth weight (0.791 ± 0.024 kg) and weighed daily. At PND0, piglets were sacrificed within their first 12 h of life and were allowed to suckle colostrum. At PND2, 5, and 10 (corresponding to 10 days, 1 and 2 months of age in Humans according to Sangild et al. (2013), piglets were separated from the sow after suckling, kept in a warm environment and sacrificed after 1 h. Six adult mini-pigs (14–16 months of age, 2 females and 4 males) were sacrificed after an overnight fast. In the second experiment, sixteen other mini-pigs (7 males and 9 females, birth weight 0.784 ± 0.026 kg, *n* = 5 for IF and *n* = 11 for DL-IF) were fed two different formulas (IF 118 kcal/100 ml and DL-IF 120 kcal/100 ml, Table 1 for fatty acid composition) from birth to PND10 in 10 meals per day using automated milk replacer feeders as previously described (Morise et al., 2009; Lemaire et al., 2018). Since passive immunity is normally acquired through colostrum and could not be acquired in these animals, we supplemented them with immunoglobulins (Ig) for 48 h. Immediately after birth and within the next 24 h later, piglets were force-fed with 10 ml of an Ig solution (0.09 g/ml). Immunoglobulins were further added to the formulas for 48 h (19 g Ig/L). The amount of formula provided to piglets was based on their body weight (346 kcal/kg body weight ^0.75^), corresponding to *ad libitum* feeding since an average 3% refusal was recorded every day. Daily formula intake was not different between the two groups (IF: 224 ± 14 ml/d and DL-IF: 217 ± 14 ml/d, *p* > 0.05). Final body weight at PND10 was not different between groups (IF: 1.01 ± 0.03 kg and DL-IF: 1.01 ± 0.06 kg, *p* > 0.05). At PND10, piglets were sacrificed 1 h after their last meal.

**TABLE 1:** Fatty acid composition (% of total fatty acid) of infant formula (IF) and infant formula formulated with dairy lipids (DL-IF).

	IF	DL-IF
C6:0	0.03	0.6
C8:0	0.02	0.5
C10:0	0.02	1.2
C12:0	0.1	1.6
C14:0	0.4	5.6
C14:1 n-5	0.0	0.5
C15:0	0.0	0.5
C16:0	31.9	20.6
C16:1 n-9	0.0	0.1
C16:1 n-7	0.09	0.9
C18:0	4.1	5.9
C18:1 n-9	46.7	44.2
C18:1 n-7	1.8	1.7
C18:2 n-6	13.3	14.7
C18:3 n-6	0.0	0.0
C18:3 n-3	1.0	1.2
C20:0	0.2	0.1
C20:1 n-9	0.2	0.2
C20:2 n-6	0.0	0.0
C20:4 n-6	0.0	0.0
C22:6 n-3	0.0	0.0

Sacrifice was performed by exsanguination after electronarcosis. Blood was sampled at exsanguination and immediately placed in EDTA K3 15% and aprotinin 250KIU tubes and kept on ice until centrifugation, which was performed within 30 min. Plasma was obtained by centrifugation (2500g, 10 min, 4°C) and further acidified by adding freshly prepared diluted (at 10 mg/ml) PMSF solution (10 µl of PMSF solution/ml plasma) and HCl 1N (50 μl/ml plasma) for acylated ghrelin analysis whereas total ghrelin was assayed in non-acidified plasma. Both acidified and non-acidified plasmas were stored at −80°C until analysis. The stomach was dissected and kept at 4°C during the whole sampling procedure. The gastric content was removed and a 1–2 g sample immediately frozen in liquid nitrogen and stored at −80°C for further fatty acid composition analysis. Gastric tissue was rinsed with ice-cold PBS. A 100 mg antral segment was placed in RNA-later at 4°C for 24 h then at −20°C for further qPCR analysis. A 200 mg segment was flash-frozen in liquid nitrogen and stored at −80°C for fatty acid composition analysis. A 5-cm^2^ antral segment was placed in 4% buffered paraformaldehyde at room temperature for 24 h, rinsed twice in 70% ethanol 1 h-bath and kept in 70% ethanol at 4°C until processing in paraffin blocks, in which 7 µm sections were made. The whole hypothalamus was dissected and immediately flash-frozen and kept at −80°C until qPCR.

### Plasma ghrelin analysis

Total and acylated ghrelin plasma levels were analyzed using Human RIA kits (GHRT-89HK and GHRA-88HK respectively, Merck KGaA, Darmstadt, Germany) validated for pigs (Woliński et al., 2014).

### Immuno-histochemistry

After rehydration, the sections were incubated in Tris-EDTA (pH 7.4) for 1 h at 95°C to retrieve antigenicity. After rinsing 3 times for 5 min with PBS, non-specific staining was blocked with a commercial blocking solution (10% goat serum, Tyramide SuperBoost™ kit, ref B40923, Thermo Fischer Scientific, Waltham, MA, United States) and sections were exposed for 1.5 h to a rabbit anti-acylated ghrelin antibody (1:50, ref GHS11-A, Alpha Diagnostic, San Antonio, Texas, United States) at room temperature. They were then rinsed 3 times (5 min) in PBS and the signal was amplified using the Tyramide SuperBoost™ kit following the manufacturer instructions. Sections were washed again with PBS and cover-slipped with Fluoroshield mounting medium (ref 104139, Abcam, Cambridge, United Kingdom). They were then examined with a fluorescence microscope (Eclipse E400, Nikon Instruments France, Champigny-Sur-Marne, France) attached to a digital camera (Digital Still DXM 1200, Nikon Instruments France). The whole section of the tissue was scanned using digital slide scanner (Nanozoomer 2.0-RS, Hamamatsu Photonics France SARL, Massy, France). Recorded files were analysed using the software ImageJ (https://rsb.info.nih.gov/ij/) for automatic determination of the number of ghrelin-labelled cells per area of mucosa.

### qPCR

qPCR on antral tissue and hypothalamus was performed as already described (Menneson et al., 2019; Arnaud et al., 2020). For hypothalamus, 100 mg of homogenate of the whole hypothalamus was used for RNA extraction. Primers used are described in Table 2. For each gene in the antrum, transcript level was normalized to the geometric mean transcript level of 2 reference genes (*b2m*, coding beta2 microglobulin protein and *rplp,* coding ribosomal protein, lateral stalk subunit P1). In the hypothalamus, *pgk1* (coding phosphoglycerate kinase 1) reference gene was used. The 2-ΔΔCt method was used and results are expressed as fold-change (FC) compared to PND 0 or the IF-fed group.

**TABLE 2:** Primer sequences.

	Forward	Reverse	Accession number	Efficiency (%)
*Ghrl*	Caa​gaa​gcc​agc​agc​caa​ac	gaa​gcc​agg​tga​gcc​ctt​ag	NM_213807.2	98.1
*Goat*	Ctg​ggc​tct​tca​aac​tca​cc	gtc​tgc​atc​agg​gac​aaa​ac	NM_001190423.1	102.1
*pcsk1/3*	aca​ggg​gag​aca​agg​aaa​gg	tga​tgg​aga​tgg​tgt​aga​tgc	NM_214038.1	103.6
*ghsr1a*	cagggaccagaaccacaa	aag​agg​aca​aag​gac​acg​ag	NM_214180.1	95.6
*b2m*	aaa​cgg​aaa​gcc​aaa​tta​cc	atc​cac​agc​gtt​agg​agt​ga	NM_213978	97.5
*Rplp*	cct​ttc​ctc​agc​tgc​cca​cta	cgt​gca​aaa​tga​ggg​cag​ag	NM_001129964.2	98.8
*pgk1*	aga​taa​cga​aca​acc​aga​gg	tgt​cag​gca​tag​gga​tac​c	NM_001099932.2	102.0

### Fatty acid analysis

Solvents were purchased from Sigma-Aldrich (Saint-Quentin Fallavier, France) or Thermo Fisher Scientific (Elancourt, France). Lipids from antrum and gastric content were extracted using a mixture of dimethoxymethane/methanol (4:1 v/v) after homogenization with an Ultra-Turrax (Lemarié et al., 2015). Heptanoic acid (C7:0) and heptadecanoic acid (C17:0) were added as internal standards in order to quantify C8:0 and C10:0 or longer chain fatty acids, respectively. The total lipid extracts were deposited on silica-impregnated plates and solvents were slowly air-dried at room temperature to ensure the recovery of volatile short and medium chain fatty acids (Lemarié et al., 2016). The silica-collected lipids were then saponified for 30 min at 70°C with 1 ml of 0.5 M NaOH in methanol and methylated for 30 min at 70°C with 1 ml boron trifluoride (14% in methanol). Fatty acid methyl esters (FAMEs) were extracted by adding 0.5 ml hexane and 6 ml NaCl 0.9% (in deionized water). The hexane phase was directly analyzed by gas chromatography coupled to mass spectrometry using an Agilent Technologies 7890N gas chromatograph (Bios Analytic, Toulouse, France) with a bonded silica capillary column (60 m × 0.25 mm; BPX 70, SGE, Villeneuve-St-Georges, France), with a polar stationary phase of 70% cyanopropylpolysilphenylene-siloxane (0.25 mm film thickness). Helium was used as carrier gas (average velocity 36 cm/s). Injection volumes were from 0.1 to 2 µl depending on the tissue, in splitless mode at 250°C. The oven temperature program started at 45°C for 3 min. Then, the temperature ramped firstly at 10°C/min to 150°C, then at 4°C/min to 260 C and was held at 260°C for 2 min. Mass detection data were obtained in full scan mode with a mass range of m/z 50–550 atomic mass unit. The National Institute of Standards and Technology database (version 2.01) was used for FAMEs identification. Peak integration was performed with MassHunter Workstation Software Qualitative Analysis Version B.07.00 (Agilent Technologies) (Guillocheau et al., 2020) and results were expressed as % of total fatty acids or as mg/g of tissue after calculation with reference to the internal standards.

### Statistical analysis

Data are presented as means ± sem. Data were analyzed using one-way ANOVA followed by Tukey test when appropriate to test differences between the 5 ages (PND0, 2, 5, 10, and adults) or *t*-test to test differences between dietary groups (IF versus DL-IF). Differences were considered significant for *p* ≤ 0.05.

## Results

### Post-natal development of the ghrelin system

**FIGURE 1:**
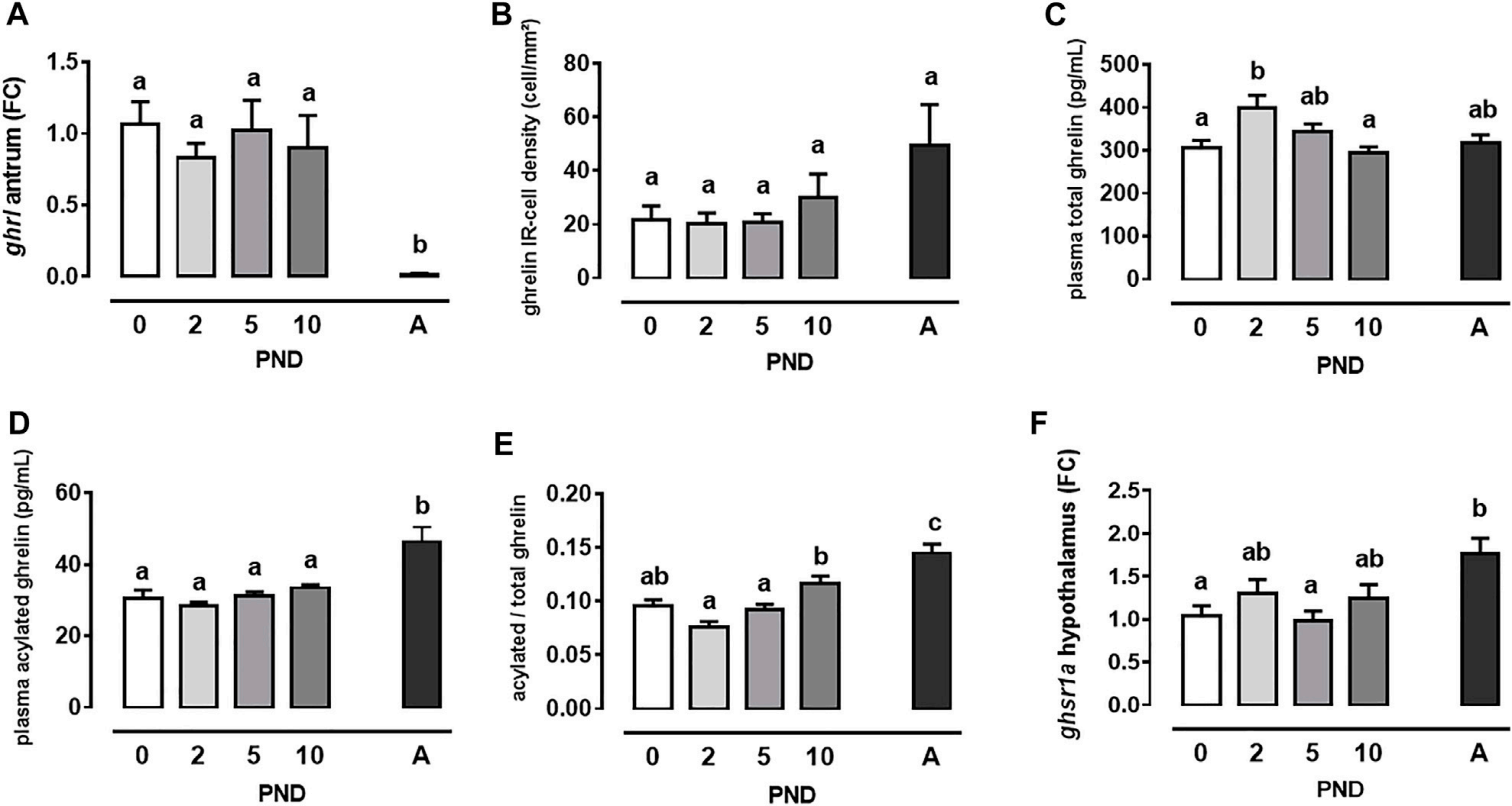
Neonatal development of the ghrelin system in mini-pigs. Pre-pro-ghrelin (*ghrl*) mRNA levels in the antrum **(A)**, density of acylated ghrelin immuno-reactive (IR) cells in the antrum **(B)**, total **(C)** and acylated **(D)** ghrelin plasma level, plasma acylated-to-total ghrelin ratio **(E)** and ghrelin receptor (*ghsr1a*) mRNA levels in the hypothalamus were measured in neonatal suckled mini-pigs (post-natal days (PND) 0–10) and in adult ones (A). Data are means +/− sem. Means with different letters are significantly different (*p* ≤ 0.05, One-way ANOVA followed by Tukey test, *n* = 9 per group during the post-natal period and *n* = 6 for adults).

To describe the post-natal development of the ghrelin system in mini-pigs, we first measured mRNA level of *ghrl* in the antrum of suckling mini-pigs from PND0 to PND10 and in adult mini-pigs. During the first 10 days of life, *ghrl* level did not vary (*p* > 0.05). However, a dramatic decrease of *ghrl* mRNA level was noted between the neonatal and the adult life (*p* < 0.0001, Figure 1A). At the proteic level, the density of acylated ghrelin-immuno-reactive (IR) cells in the antrum did not vary during the neonatal period (PND0 to PND10) and was not different from that of adult mini-pigs (*p* > 0.05, Figure 1B and Supplementary Figure 1). We next measured total and acylated plasma ghrelin levels, 1 h after the last suckling episode for neonates and after an overnight fast in adult mini-pigs. Total plasma ghrelin was greater at PND2 compared to PND0 and 10 (*p* = 0.02 and *p* = 0.008, respectively, Figure 1C). Acylated ghrelin plasma levels did not vary during the PND0-PND10 period but was greater in adult mini-pigs compared to neonates (*p* < 0.0001 for all comparisons to adults, Figure 1D). Consequently, the acylated-to-total ghrelin ratio in the plasma significantly increased with age (PND2 vs. PND10, *p* = 0.0002, PND2 vs. A, *p* < 0.0001, PND10 vs. A *p* = 0.04, Figure 1E). Finally, at the hypothalamus level, we measured ghrelin receptor (*ghsr1a*) mRNA levels, which did not vary during the neonatal period but was greater in adult mini-pigs compared to neonates at 0 and 5 days of age (*p* = 0.02 and 0.008, respectively, Figure 1F).

**FIGURE 2:**
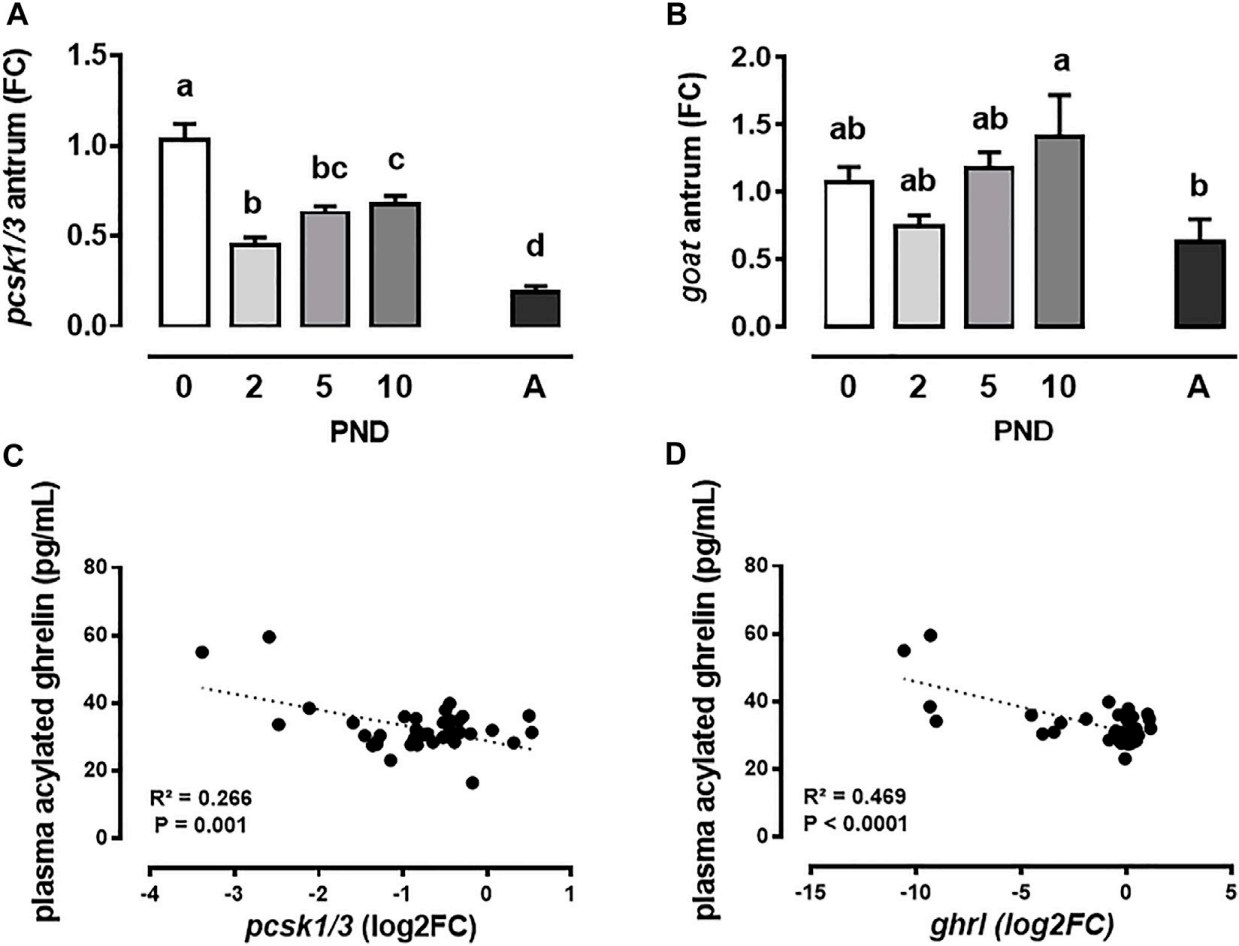
Neonatal development of ghrelin maturation enzymes in mini-pigs. Convertase 3 (*pcsk*1/3) **(A)** and ghrelin-O-acetyl transferase (*goat*) **(B)** mRNA levels in the antrum were measured in neonatal suckled mini-pigs (post-natal days (PND) 0–10) and in adult ones **(A)**. Pearson correlation between *pcsk*1/3 **(C)** or *goat* **(D)** mRNA levels in the antrum and plasma acylated ghrelin.

To further explore the ghrelin system, we evaluated the mRNA levels of genes encoding enzymes involved in ghrelin maturation, i.e., the prohormone convertase 1/3 (*pcsk1/3*) responsible for the proteolytic cleavage of pre-proghrelin to ghrelin and GOAT (*goat*) which then catalyzes the acylation of ghrelin. The level of *pcsk1/3* mRNA decreased by 50% between PND0 and PND2 (*p* < 0.0001, Figure 2A) then increased until PND10 (PND2 vs. PND10, *p* = 0.05, Figure 2A). It then decreased sharply between PND10 and adult mini-pigs (PND10 vs. A, *p* < 0.0001, Figure 2A). The level of *goat* mRNA did not vary significantly in the antrum from PND0 to PND10 but was lower in adult compared to PND10 mini-pigs (*p* = 0.05, Figure 2B). Interestingly, the level of plasma acylated ghrelin correlated negatively with *pcsk1/3* (Figure 2C) and *ghrl* (Figure 2D) mRNA levels in the antrum, but not with *goat* mRNA level (data not shown). Noteworthy, these significant correlations were mainly driven by adult data since it was no more significant when only the neonatal period was considered (data not shown).

### Effect of dietary MCFA on ghrelin system

Since acyl modification of ghrelin is essential for its function, with octanoylation being the most common one, and since maternal milk is source of MCFA, we sought to evaluate if dietary MCFA played a role in the post-natal development of the ghrelin system.

We first analyzed the fatty acid composition of gastric content in a subset of neonatal piglets (*n* = 4 per age group) at different time-points as a proxy of sow milk composition to investigate the evolution with age of dietary MCFA intake in sow-fed piglets. All the fatty acids concentrations increased with age in the gastric content (Table 3). This included C8:0 and C10:0, whose concentration within the gastric content increased by 3.9- and 16.9-fold, respectively, between PND0 and PND10 (Table 3). We next evaluated antrum fatty acid composition to assess if the changes in gastric content MCFA composition resulted in significant changes at the antral tissue level. However, during the neonatal period, no variation in fatty acid concentration was observed in the antrum, except for C15:0 whose concentration decreased with age (Table 4). During the neonatal period (PND 0–10), C10:0 quantity in the antrum was negatively correlated to the acylated-to-total ghrelin ratio and *goat* mRNA level (r = −0.497, *p* = 0.05 and r = −0.504, *p* = 0.047, respectively).

**TABLE 3:** Fatty acid composition (µg/g) of gastric content of suckled piglets.

	PND0	PND2	PND5	PND10	Age effect
C8:0	37 ± 16 a	43 ± 20 ab	111 ± 38 ab	144 ± 22 b	0.03
C10:0	54 ± 16 a	280 ± 106 a	930 ± 87 b	913 ± 120 b	<0.0001
C12:0	56 ± 10 a	278 ± 77 b	628 ± 19 c	625 ± 42 c	<0.0001
C14:0	753 ± 178 a	4958 ± 1052 b	8380 ± 233 c	7808 ± 635 c	<0.0001
C14:1 n-5	16 ± 5 a	216 ± 69 a	521 ± 65 b	484 ± 73 b	0.0002
C15:0	98 ± 27 a	407 ± 56 c	302 ± 36 bc	210 ± 21 b	0.0005
C16:0	8731 ± 1787 a	46322 ± 7208 b	65709 ± 2371 c	56678 ± 4631 bc	<0.0001
C16:1 n-9	368 ± 53 a	1447 ± 126 b	1467 ± 218 b	1156 ± 124 b	0.0005
C16:1 n-7	1023 ± 222 a	11913 ± 2659 b	20886 ± 1581 c	17734 ± 2039 bc	<0.0001
C17:0	248 ± 65 a	975 ± 143 c	683 ± 65 bc	532 ± 46 ab	0.0007
C18:0	2013 ± 324 a	9952 ± 870 b	10111 ± 876 b	9543 ± 834 b	<0.0001
C18:1 n-9	8322 ± 1053 a	52164 ± 3357 b	73897 ± 3021 b	58894 ± 2557 b	0.004
C18:1 n-7	841 ± 95 a	4696 ± 447 b	6581 ± 755 b	5197 ± 1649 b	0.006
C18:2 n-6	6701 ± 1314 a	28487 ± 3357 b	29243 ± 3021 b	28188 ± 2557 b	0.0001
C18:3 n-3	561 ± 113 a	3060 ± 501 b	3673 ± 358 b	3616 ± 393 b	0.0002
C20:0	58 ± 10 a	289 ± 31 b	312 ± 27 b	295 ± 29 b	<0.0001
C20:1 n-9	69 ± 9 a	455 ± 48 ab	681 ± 103 b	682 ± 234 b	0.02
C20:2 n-6	85 ± 28 a	493 ± 29 b	522 ± 89 b	693 ± 60 b	<0.0001
C20:3 n-6	78 ± 19	220 ± 59	256 ± 46	252 ± 50	n.s.
C20:4 n-6	285 ± 51 a	1212 ± 95 b	1329 ± 153 b	955 ± 205 b	0.0008
C22:0	30 ± 8 a	158 ± 23 b	148 ± 13 b	151 ± 18 b	0.0003

Data are means +/− sem. Means with different letters are significantly different (*p* ≤ 0.05, One-way ANOVA followed by Tukey test, *n* = 4 per group). n.s.: not significant.

**TABLE 4:** Fatty acid composition (µg/g) of antrum during the neonatal period.

	PND0	PND2	PND5	PND10	Age effect
C8:0	33 ± 12	46 ± 16	16 ± 6	23 ± 3	n.s.
C10:0	39 ± 12	61 ± 17	26 ± 8	33 ± 6	n.s.
C12:0	194 ± 52	269 ± 79	108 ± 33	140 ± 16	n.s.
C14:0	205 ± 27	460 ± 131	193 ± 51	356 ± 169	n.s.
C14:1 n-5	9 ± 2	30 ± 9	13 ± 5	26 ± 10	n.s.
C15:0	53 ± 9 a	56 ± 11 a	16 ± 6 b	19 ± 5 b	0.005
C16:0	2492 ± 344	4586 ± 1035	2291 ± 605	4245 ± 1668	n.s.
C16:1 n-9	164 ± 23	195 ± 28	76 ± 20	133 ± 52	n.s.
C16:1 n-7	327 ± 52	850 ± 286	398 ± 107	860 ± 502	n.s.
C17:0	233 ± 53	276 ± 110	75 ± 13	100 ± 17	n.s.
C18:0	1287 ± 225	1744 ± 307	1100 ± 393	1924 ± 611	n.s.
C18:1 n-9	2371 ± 445	2296 ± 932	1077 ± 647	951 ± 2293	n.s.
C18:1 n-7	1033 ± 97	638 ± 116	356 ± 110	716 ± 289	n.s.
C18:2 n-6	1033 ± 292	2296 ± 595	1077 ± 305	951 ± 344	n.s.
C18:3 n-6	23 ± 6	45 ± 17	11 ± 2	20 ± 9	n.s.
C18:3 n-3	59 ± 23	161 ± 54	60 ± 17	136 ± 82	n.s.
C20:0	21 ± 5	30 ± 6	12 ± 4	21 ± 8	n.s.
C20:1 n-9	22 ± 6	35 ± 11	25 ± 9	52 ± 27	n.s.
C20:2 n-6	18 ± 7	54 ± 15	28 ± 11	46 ± 22	n.s.
C20:3 n-6	50 ± 12	61 ± 22	41 ± 17	62 ± 9	n.s.
C20:4 n-6	993 ± 201	1069 ± 274	713 ± 286	894 ± 52	n.s.
C20:5 n-3	14 ± 4	25 ± 10	15 ± 6	21 ± 2	n.s.
C22:4 n-6	128 ± 30	150 ± 43	105 ± 54	130 ± 13	n.s.
C22:5 n-6	63 ± 16	33 ± 11	21 ± 13	27 ± 4	n.s.
C22:5 n-3	44 ± 8	92 ± 29	71 ± 34	102 ± 16	n.s.

Data are means +/− sem. Means with different letters are significantly different (*p* ≤ 0.05, One-way ANOVA followed by Tukey test, *n* = 4 per group). n.s.: not significant.

**FIGURE 3:**
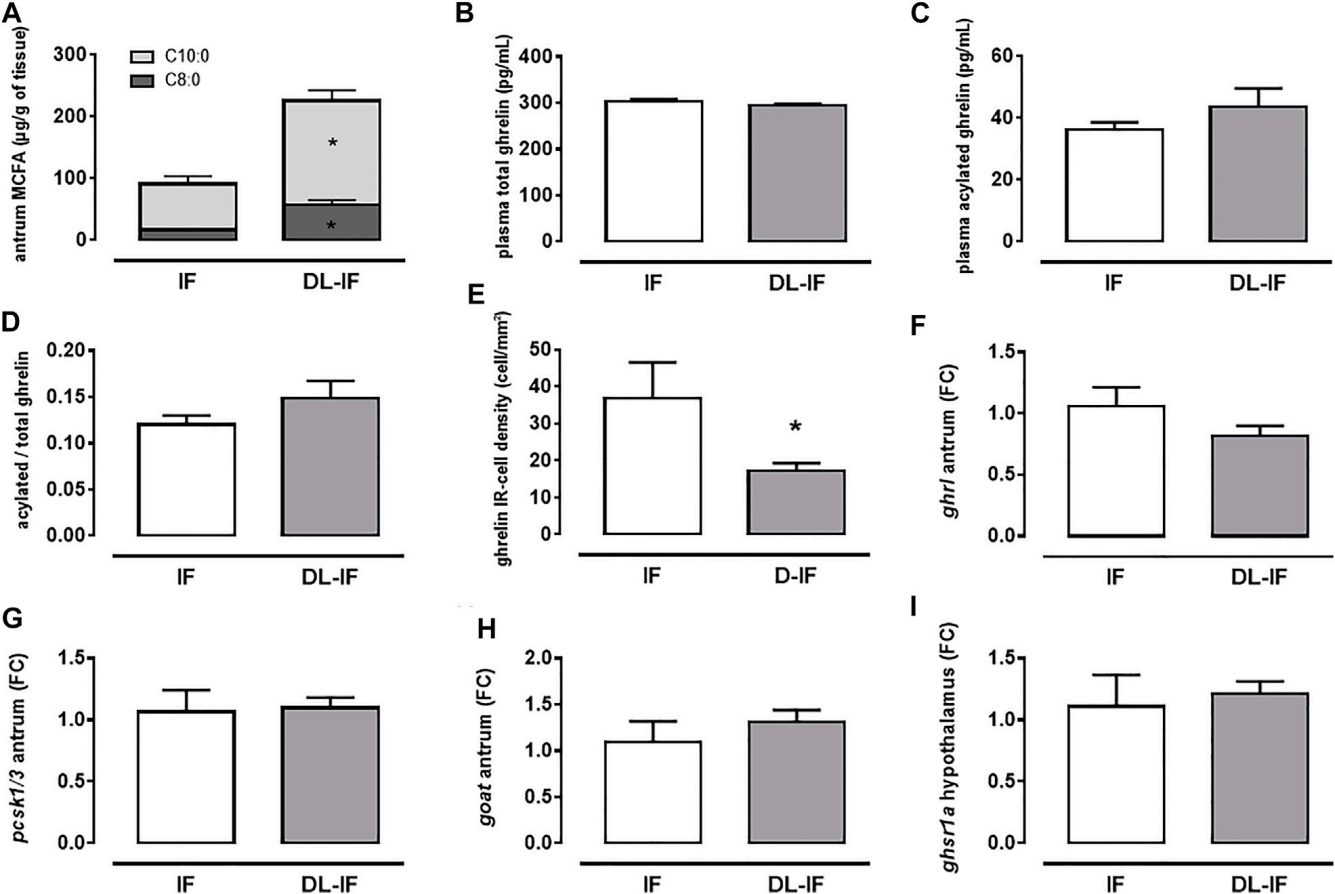
Impact of dietary MCFA formula on the ghrelin system. Medium chain fatty acids (MCFA, C8:0, and C10:0) content in the antrum **(A)**, total **(B)** and acylated **(C)** ghrelin plasma level, plasma acylated-to-total ghrelin ratio **(D)**, density of acylated ghrelin immuno-reactive (IR) cells in the antrum **(E)**, pre-pro-ghrelin (*ghrl*) **(F)**, convertase 3 (*pcsk*1/3) **(G)** and ghrelin-O-acetyl transferase (*goat*) **(H)** mRNA levels in the antrum and ghrelin receptor (*ghsr1a*) mRNA levels in the hypothalamus **(I)** were measured in mini-piglets fed infant formulas formulated with plant lipids (IF) or with a mix of plant and dairy lipids (DL-IF) to modulate MCFA dietary levels. Data are means +/− sem. *: *p* ≤ 0.05, Student *t*-test (*n* = 5 and 11 for IF and DL-IF respectively).

We next assessed if the level of dietary MCFA in the neonatal diet could impact the development of the ghrelin system. We used two infant formulas, one formulated with plant and dairy lipids (DL-IF), providing MCFA and one formulated uniquely with plant lipids, thus with very low level of MCFA (Table 1). We fed mini-pigs for 10 days with these two formulas before evaluating their ghrelin system. Feeding IF or DL-IF for 10 days resulted in a significant difference in antrum MCFA content (*p* = 0.004, Figure 3A). Despite this difference, the ghrelin system of piglets was poorly affected. Total, acylated and acylated-to-total plasma ghrelin was similar between IF-fed and DL-IF fed piglets (Figures 3B–D). Levels of *ghrl* (Figure 3F), *pcsk1/3* (Figure 3G) and *goat* (Figure 3H) mRNA in the antrum and of *ghsr1a* mRNA in the hypothalamus (Figure 3I) did not vary with diet. However, the density of acylated ghrelin-IR cells in the antrum was greater in piglets fed IF compared to DL-IF (*p* = 0.01, Figure 3E and Supplementary Figure 1).

## Discussion

Our study in an animal model close to humans in terms of gastro-intestinal physiology and eating behavior (Roura et al., 2016) shows changes in plasma ghrelin levels during the early neonatal period and compared to adulthood: plasma total ghrelin peaked at PND2 but was stable thereafter while plasma acylated ghrelin did not vary during the neonatal period but was greater in adult mini-pigs that in neonatal ones. In two studies using the same animal model, plasma total ghrelin did not significantly change with post-natal age (between PND 0 and 28 (Willemen et al., 2013) or PND 1 and 7 (Woliński et al., 2014). However, in both studies, blood sampling was performed 30–35 min after suckling while we waited 1 h, which might account for the difference in the post-natal kinetic although it remains unclear if ghrelin level correlates with fasting time in neonates (Bellone et al., 2006; James et al., 2007). Our data follow the same pattern than what has been described in humans, although with a different kinetic probably due to inter-species differences. Indeed, several studies in humans reported an increase in plasma total ghrelin during the first neonatal months with a peak likely occurring around 24–30 months of age (Soriano-Guillén et al., 2004; Savino et al., 2005, 2006; Yokota et al., 2005) followed by a decrease in children and teenagers (Whatmore et al., 2003; Soriano-Guillén et al., 2004; Yokota et al., 2005; Wilasco et al., 2012). Conversely, data on the change with age of acylated ghrelin in plasma are scarcer and less consistent, probably due to major differences in study protocols: in two studies, acylated ghrelin plasma level did not vary between birth and 1-week of life (Yokota et al., 2005) or between 0 and >60 months of age (Wilasco et al., 2012) but a third study indicated an increase in acylated ghrelin plasma level between 3 and 6 months of age (de Fluiter et al., 2021). Later in life, one study reported a greater level of acylated ghrelin in children plasma (average age 8 years) compared to neonate one (cord blood samples) (Bellone et al., 2012), as observed in our study.

The changes in plasma acylated ghrelin did not correlate with the density of acylated ghrelin IR cells in the stomach but correlated negatively with *ghrl* and *pcsk1/3* expression at the gastric level. Total ghrelin plasma level did not correlate with any of the gene expression studied in the antrum. Importantly, we studied gene transcript level and not protein expression (or activity where appropriate) at the gastric level, which constitutes a limitation of our study. A recent study in older piglets described an increase in *ghrl* but no variation of *goat* or *pcsk1/3* expressions between PND 14 and 42 (Trevisi et al., 2021). However, timepoints at younger ages as well as corresponding plasma ghrelin levels like in our study were not reported. In mouse models, discrepancies between ghrelin-related gene expression and plasma ghrelin level variations with post-natal age have also been documented (Liu et al., 2002; Sun et al., 2020). Noteworthy, plasma ghrelin concentration depends on ghrelin secretion from stomach to plasma, as well as ghrelin clearance, which are regulated by many factors (fatty acid responsive receptor GPR120, acyl-protein thioesterase 1 activity in the plasma, ghrelin reactive immunoglobulins) (Lemarié et al., 2018). Thus, a direct correlation between gene expressions and plasma levels is difficult to establish. Another factor possibly influencing plasma total or acylated ghrelin during the neonatal period is milk ghrelin content. In humans, an increase in ghrelin level between colostrum, transient and mature milk has been observed (Fields et al., 2016). In pig, only one study evaluated milk ghrelin level during the first week of lactation. No variation in milk ghrelin level was observed. Yet, a strong positive correlation between colostrum and piglet plasma total ghrelin levels was observed at PND 1 but not with milk at later time points (PND 2 and 7) (Woliński et al., 2014), suggesting the development of the piglet own ghrelin system after PND 2 and independency from milk ghrelin in plasma ghrelin levels. Yet a role of milk ghrelin in intestinal development has been suggested based on the observation of increased intestinal epithelial cell proliferation in piglets orally supplemented with ghrelin every 8 h from birth to PND 6 (Słupecka et al., 2012). However, since sow milk ghrelin level does not vary with lactation stage (Woliński et al., 2014), we could speculate that the ghrelin transient peak we observed in plasma at PND 2 is involved in the increase in crypt depth and villus length observed between PND 2 and 7 in piglets (Arnaud et al., 2020). Noteworthy, in our study, plasma total, but not acylated, ghrelin did not vary during the neonatal period. Unfortunately, the acylation state of the exogenous ghrelin administrated to piglets was not reported in the study by Slupecka et al. Therefore, the physiological consequences of the age-related differences in plasma acylated versus non-acylated ghrelin reported in our study on the gastro-intestinal tract warrant further investigations.

The ghrelin system involves ghrelin secretion and circulation and its receptor at the central level. To our knowledge, our study is the first one that evaluated the post-natal expression of the ghrelin receptor (*gshr1a*) at the hypothalamic level in the porcine model. Only data in mice were available so far: no changes were observed between PND 6 and adult life, except for a significant decrease at PND14 (Steculorum et al., 2015; Sun et al., 2020). Mice, like rats, are altricial species and as such, considered immature at birth compared to humans or pigs. Transcriptome correlations combining all somatic organs in different species revealed that the immediate post-natal period in mice (from birth to PND14) is analogous to the third trimester of gestation in humans (Cardoso-Moreira et al., 2019). On the other hand, gut maturation is achieved during the post-natal period, before the weaning transition occurring at PND21-28 in pigs, closely to the timing of infant gut maturation (Sangild et al., 2013). Getting data in pigs is therefore valuable as a proxy of the human situation. An increase in *ghsr* mRNA level was observed between the neonatal piglets and adult pigs. We have to acknowledge that adult pigs were sampled after an overnight fast while piglets were fasted only 1 h. Whether *gshr* mRNA level is sensitive to the length of the fasting period remains to be evaluated. During the neonatal period, no variation of *gshr* expression was observed. However, we did not evaluate ghrelin sensitivity in our piglet model, either through eating behavior tests or neuronal response (neuronal firing, activation of intra-cellular signaling, etc.) to ghrelin exogenous injection. This was beyond the scope of the study but such studies would be important to evaluate the actual role of ghrelin as eating behavior regulator and/or as hypothalamic neuronal development player as demonstrated in mice (Steculorum et al., 2015). Interestingly, the daily suckling frequency in piglets follows an exponential curve with a sharp increase of the number of suckling bouts after PND 2 until PND 8, where the maximum number is reached, and a decrease thereafter (Puppe and Tuchscherer, 2000). The involvement of the progressive increase of the plasma acylated to total ghrelin ratio we observed from PND 2 to 10 in this suckling pattern warrants further investigations.

Another factor that could influence ghrelin system post-natal evolution is the neonatal diet. Several reports indicated that the maternal diet during both gestation and lactation can affect plasma ghrelin levels (Słupecka et al., 2016; Kisioglu and Nergiz-Unal, 2020), gene expression at the gastric level (Sun et al., 2020) or even ghrelin receptor expression and/or sensitivity to ghrelin at the central level (Rossetti et al., 2020). Data testing the impact of nutrition after birth attest that this period is a window of nutritional modulation of the ghrelin system. Indeed, ghrelin plasma level is greater in formula-fed than breast-fed infants (Savino et al., 2005; de Fluiter et al., 2021), although one study contradicted these results (Savino et al., 2006). In animal models, only neonatal overnutrition obtained by manipulating litter size has been studied so far: it reduced plasma acylated and total ghrelin levels (Collden et al., 2015), increased acylated ghrelin gastric tissue content (Du et al., 2015) as well as *goat* mRNA levels (Collden et al., 2015) while at the central level, it reduced hypothalamic *ghsr* mRNA level and ghrelin sensitivity during the neonatal period in mouse models (Collden et al., 2015). In our study, we evaluated the role of dietary MCFA on the neonatal development of the ghrelin system. MCFA levels in the antrum did not vary with post-natal age despite changes in MCFA content of gastric contents in suckling piglets. Nonetheless, C10:0 level in the antrum was negatively correlated to acylated-to-total ghrelin ratio in the plasma and to *goat* mRNA expression. Thus, considering that MCFA is provided by breast-milk during the neonatal period, we sought to evaluate if the level of dietary MCFA impact the ghrelin system development and would be an important factor to consider when formulating milk substitutes for neonates. Our DL-IF formulated with plant and dairy lipids had higher levels of MCFA compared to IF (C6:0 + C8:0 + C10:0 = 2.3% of total fatty acids vs. 0.07% in IF) and compared to gastric contents of suckled piglets (0.07, 0.08, 0.23, and 0.23% of total fatty acids at PND0, 2, 5, and 10, respectively). DL-IF feeding resulted in increased levels of C8:0 and C10:0 in the antrum (x 2.7), while feeding the IF formula resulted in levels similar to that of suckling piglets of the same age. However, despite this increase in C8:0 and C10:0 in the antrum of DL-IF piglets, few differences were observed in the ghrelin system between IF and DL-IF fed piglets. Only the density of acylated ghrelin-IR cells was reduced in DL-IF fed piglets. This 53% decrease in the density of acylated ghrelin-IR cells in the stomach is in line with the 33% decrease in total ghrelin-IR cells observed in rats fed a diet containing 8% (total fatty acids) of C8:0 during 3 weeks, although in the same study, *ghrl* expression was greatly reduced unlike in our (Lemarié et al., 2015). The low impact of dietary MCFA in our study while some effects of dietary C8:0 have been reported might be attributed to the relatively low level of supplementation in our study. Indeed, the effects of dietary C8:0 on the ghrelin system was observed in animal models with diets containing C8:0 levels greater than 8% of fatty acids (Nishi et al., 2005a; Lemarié et al., 2015; Kaur et al., 2020) while our DL-IF contained only 2.3% of MCFA, mainly provided by C10:0 (1.2%) while C8:0 accounted for 0.5% of total fatty acids. Sow milk contains 0.25% of MCFA (sow milk sampled at PND 21, Rioux, personal communication). Our DL-IF already provided 10 times more MCFA than the physiological level of suckled piglets and we did not want to go beyond this level. Another point to consider is that formula-fed piglets were sampled in a fed state (1 h after their last meal), which might have dampened MCFA effect. A positive impact of dietary MCFA on plasma acylated ghrelin level was observed in the fasted state in chicken (Yamato et al., 2005), while in mice not controlled for their feeding state, no effect of MCFA on circulating ghrelin level was observed (Kaur et al., 2020). However, studying food-deprived piglets would have been non-physiological since piglets are barely fasted as they usually suckle more than 30 times per day (Puppe and Tuchscherer, 2000). Lastly, we studied only one timepoint (PND10) to evaluate the impact of dietary MCFA on the ghrelin system; some earlier effects could have been missed.

In conclusion, our data provide evidences that the ghrelin system display a precise post-natal development in piglets that resembles what has been observed in humans at the plasma level. Interestingly, acylated ghrelin plasma level, which is important for ghrelin biological effects at the central level correlated to *ghrl* and *pcsk1/3* but not *goat* mRNA level in the stomach. Likewise, the acylated-to-ratio level also correlated to C10:0 (but not C8:0) antral content. Yet, modulating MCFA level through the diet only had limited effect of the ghrelin system.

## Data Availability

The original contributions presented in the study are included in the article/Supplementary Materials, further inquiries can be directed to the corresponding author.
