# Supplementary figures and images for: The ghrelin system follows a precise post-natal development in mini-pigs that is not impacted by dietary medium chain fatty-acids
Source: Front Physiol. 2022 Sep 26;13:1010586. doi: 10.3389/fphys.2022.1010586 (PMC9549131; doi:10.3389/fphys.2022.1010586)

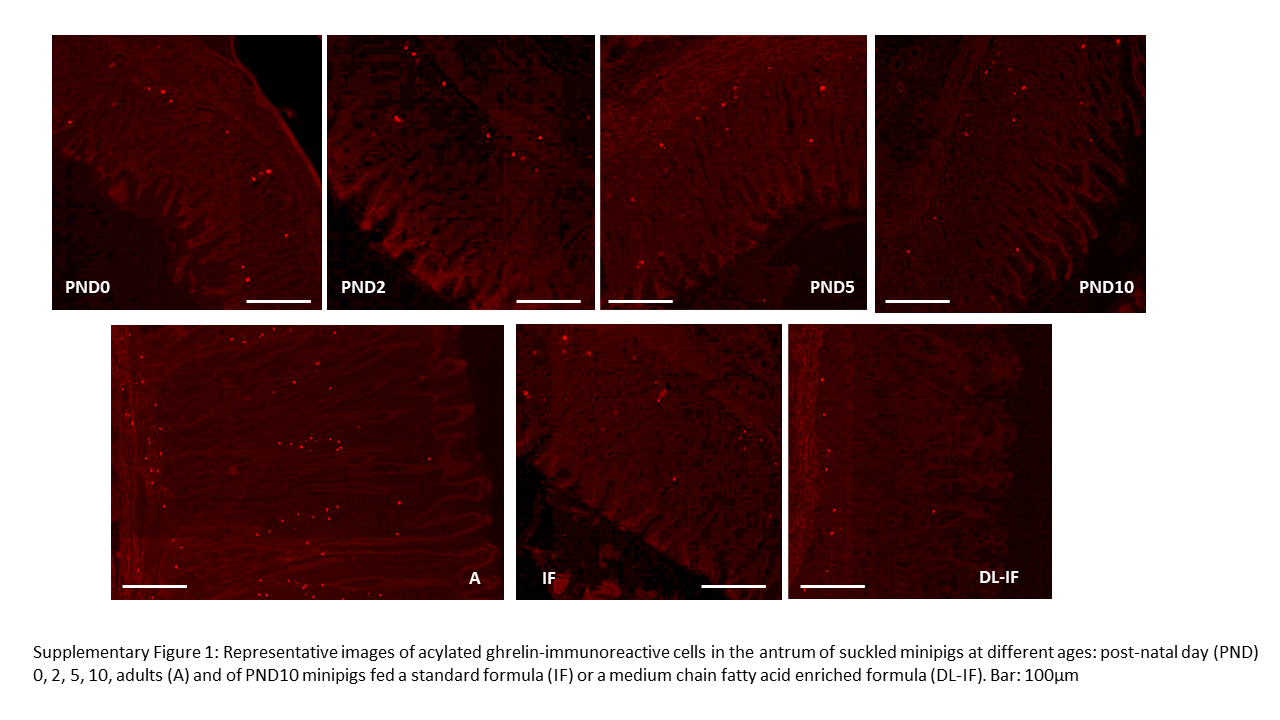

Supplement: Supplementary file 1 [file Image1.TIF]
